# Patterns in Species Persistence and Biomass Production in Soil Microcosms Recovering from a Disturbance Reject a Neutral Hypothesis for Bacterial Community Assembly

**DOI:** 10.1371/journal.pone.0126962

**Published:** 2015-05-11

**Authors:** Fen-Guo Zhang, Quan-Guo Zhang

**Affiliations:** State Key Laboratory of Earth Surface Processes and Resource Ecology and MOE Key Laboratory for Biodiversity Science and Ecological Engineering, Beijing Normal University, Beijing, China; University of Oklahoma, UNITED STATES

## Abstract

The neutral theory of biodiversity has emerged as a major null hypothesis in community ecology. The neutral theory may sufficiently well explain the structuring of microbial communities as the extremely high microbial diversity has led to an expectation of high ecological equivalence among species. To address this possibility, we worked with microcosms of two soils; the microcosms were either exposed, or not, to a dilution disturbance which reduces community sizes and removes some very rare species. After incubation for recovery, changes in bacterial species composition in microcosms compared with the source soils were assessed by pyrosequencing of bacterial 16S rRNA genes. Our assays could detect species with a proportional abundance ≥ 0.0001 in each community, and changes in the abundances of these species should have occurred during the recovery growth, but not be caused by the disturbance *per se*. The undisturbed microcosms showed slight changes in bacterial species diversity and composition, with a small number of initially low-abundance species going extinct. In microcosms recovering from the disturbance, however, species diversity decreased dramatically (by > 50%); and in most cases there was not a positive relationship between species initial abundance and their chance of persistence. Furthermore, a positive relationship between species richness and community biomass was observed in microcosms of one soil, but not in those of the other soil. The results are not consistent with a neutral hypothesis that predicts a positive abundance-persistence relationship and a null effect of diversity on ecosystem functioning. Adaptation mechanisms, in particular those associated with species interactions including facilitation and predation, may provide better explanations.

## Introduction

A major advance in ecology in the past several decades is the development of the neutral theory of biodiversity [1,2,3]. This theory has a very simplistic, and seemingly unrealistic, assumption that all species within a community are ecologically equivalent (i.e. with the same rates of birth, death, dispersal and speciation), and thus the fate of each species in its community is largely determined by drift. Models with the neutral assumption have produced predictions for a number of biodiversity patterns (such as species abundance distribution and species-area relationship) that are often highly consistent with observations, and the neutral theory has become a major null hypothesis for explaining ecological community assembly [2,4,5]. The importance of neutral processes in microbial communities has recently received much attention [6,7,8,9,10]. In many ecosystems the diversity of free-living microbes is orders of magnitude higher than that of animals and plants; but the niche space for microbes does not seem to be very high-dimensional [11,12]. Thus high functional equivalence among microbes is expected: a large number of microbial taxa may be replaceable in terms of their ecological effects on interacting species or ecosystem processes [13,14,15,16]. This can lead to two specific hypotheses: (i) the structure of microbial communities is largely governed by drift effects in ecological processes including extinction, recruitment, immigration and speciation [2,4,5,9,10] and (ii) decline of microbial diversity does not impair ecosystem functions [2,12,17,18]. The first hypothesis is relevant to the maintenance of microbial diversity, and the second involves the functions of microbial diversity. A number of experimental studies have addressed these questions, with contradictory findings. For instance, the importance of neutral assembly processes in microbial communities has been evidenced [8] or rejected [19] in experiments that investigated microbial communities colonizing sterile microcosms, where the physical distance and environmental dissimilarity among microcosms had been manipulated. Microcosm experiments that simulated microbial species loss have found both positive [20,21] and neutral effects [14,22] of diversity on ecosystem functions.

Here we consider both the maintenance and function of microbial diversity in one experimental system, to unambiguously test for the importance of neutral assembly processes in microbial communities. We worked with soil microcosms either exposed, or not, to a dilution-to-extinction disturbance. The disturbance is analogous to non-selective harvest of biomass, executed by inoculating sterile soil microcosms with serial dilutions of a suspension obtained from the same non-sterile soil, and subsequent incubating for recovery of microbial communities [23,24]. This disturbance decreases the absolute abundances of species and removes some very rare species, with little change in the physical environment. Earlier work found that microbial communities recovering from the dilution disturbance showed a reduction in species diversity, and studied the consequences of the diversity decrease for ecosystems functioning [14,20,21]. Detailed analysis of species persistence in the recovered communities has been lacking in those studies, which we suggest can infer the mechanisms of community assembly. Changes in species composition during the recovery growth may be caused by drift processes. Alternatively, it can result from operation of adaptation mechanisms, such as *K*-species (oligotrophic species) being replaced by *r*-species (copiotroph species; as release of competition by the disturbance may select for fast-growing species) [25,26,27,28]. In the present study we first tested a specific prediction of the neutral hypothesis for community dynamics during the recovery growth following the dilution disturbance: the initially more abundant species are, on average, less likely to go extinct. Absence of such a positive abundance-persistence relationship would reject the possibility that neutral processes be the predominant driver of community structure (although existence of such a relationship does not falsify either neutral or adaptation mechanisms). Then we investigated whether changes in microbial diversity in the recovered communities were associated with changes in biomass production.

## Material and Methods

### Soil Microcosms

Two soils were collected at a semiarid steppe (Xilingol, Inner Mongolia, China; 43°38’N, 116°42’E, 1100 m.a.s.l.). One soil was referred to as ‘sandy soil’, collected from a patch of sandy land with little vegetation cover (soil texture, sand; moisture content, ~6%). The other soil was referred to as ‘grassland soil’, collected from a grassland dominated by *Stipa capillata* (soil texture, silt; moisture content, ~12%). Soils were collected to a depth 15 cm, sieved to < 2 mm, and homogenized. Inner Mongolia Grassland Ecosystem Research Station, the Chinese Academy of Sciences, issued the permission for our soil collecting work at the two sites.

From each soil, one sample was frozen at -80°C (for characterization of bacterial species compositions for the source soils), one sample was stored at 4°C (for 9 days) and later used for preparing soil suspension, and the remaining was divided into 25 microcosms of 100 g (equivalent dry mass) in 250 mL Schott Duran bottles. Among the 25 microcosms, five were assigned as control microcosms (not disturbed), stored at 4°C until the ‘recovery incubation’; the other 20 microcosms were sterilized by 100 kGy gamma irradiation (Hongyisifang radiation technology Co., Ltd, Beijing, China), with soil sterility checked by enumeration of culturable bacteria with nutrient agar plates (3 g L^-1^ beef extract, 10g L^-1^ peptone, 5 g L^-1^ NaCl and 15 g L^-1^ agar). Inocula for sterilized soil microcosms were prepared by homogenizing 30 g of soil (equivalent dry mass) in 150 ml of sterile demineralized water by grinding and vortexing, followed by serial 10-fold dilution in sterile water. Sterile soil microcosms were inoculated with 5 mL of proper dilutions, creating inocula equivalent to 10^–2^, 10^–4^, 10^–6^, 10^–8^ g of non-sterile soil g^-1^ sterile soil, five replicates per treatment level. Each of the control microcosms was inoculated with 5 mL of sterile water instead of inocula. Soil microcosms were incubated for recovery at 25°C, with loose lids. Moisture content was maintained at the initial levels (6% for the sandy soil and 12% for the grassland soil) by addition of sterile water once a week. Recovery of microbial biomass was surveyed by enumerating bacteria culturable on nutrient agar plates regularly during week 5–12 for a subset of microcosms (S1 Fig). At week 12, all microcosms were sampled.

For each soil sample (either a source soil or a microcosm), three subsamples were used for DNA extraction using FastDNA SPIN Kit for Soil (MP Biomedicals, Santa Aan, CA, USA), following the manufacture’s instructions. Briefly, in a 2 mL Lysing Matrix E tube, 0.5 g of soil was mixed with 978 μL of SPB buffer and 122 μL of MT buffer. The samples were homogenized for 40 s at speed 6.0 in a Fastprep-24 (MP Biomedicals, Santa Ana, CA, USA), and centrifuged at 14,000 g for 15 min at 20°C. Supernatant was collected to a new sterile tube, mixed with 250 μL of PBS buffer, and centrifuged at 14,000 g for 5 min. Supernatant was transferred to a new sterile tube and mixed with 1 mL of Binding Matrix Suspension, and left static for 3 min at room temperature. Then 500 μL of top-layer supernatant was discarded; the remaining was re-suspended, and flowed through a SPIN Filter, then 500 μL of SEWS-M was flowed through the same filter. The filter was moved to a fresh catch tube, air-dried for 5 min at room temperature, then added with 80 μL of DNase/Pyrogen-free sterile water, and incubated for 5 min at 55°C. The catch tubes were centrifuged at 14,000 g for 1 min, and then stored at -20°C. The five sandy soil microcosms under 10^–8^ dilution treatment were excluded from subsequent analyses, for which PCR amplification was unsuccessful due to the very low quantity of template DNA.

### Bacterial Community Structure

For each soil sample, the three replicate DNA extracts were amplified, and then pooled into one sample for barcoded pyrosequencing. We amplified 16S rRNA genes with the universal primer pair 27F (5′-AGAGTTTGATCCTGGCTCAG-3′) and 533R (5′-TTACCGCGGCTGCTGGCAC-3′), with the 533R primer tagged with unique barcodes. The PCR reactions were carried out in a 20-μL volume, containing 4 μL (5×) of FastPfu Buffer, 2 μL (2.5 mM) of dNTPs, 0.4 μL (5μM) of each forward and reverse primers, 0.4 μL of FastPfu Polymerase, ~10 ng of template DNA and ddH_2_O. Samples were initially denatured at 94°C for 5 min, amplified for 10 cycles of 45 s at 94°C, 45 s at 63°C, 30 s at 72°C (with a decrease of 0.8°C every cycle, to improve PCR yield); then for another 15 cycles of 45 s at 94°C, 45 s at 55°C and 45 s at 72°C, followed by a final 10-min extension at 72°C. The PCR products were agarose gel verified, pooled and purified using the AxyPrepDNA QIAquick Gel Extraction Kit (AXYGEN). Pyrosequencing was performed on a Roche’s 454 FLX Genome Sequencer (Shanghai Majorbio Bio-pharm Technology Co., Ltd, China).

Raw sequence data were demultiplexed and screened using the QIIME sequence analysis platform [29]. Sequences were quality trimmed with the following parameters: quality score > 20, sequence length > 200, length of homopolymer < 10 and ambiguous base calls < 1; and chimeric sequences were identified using the UCHIME method [30], and removed. The quality sequences were assigned to soil samples based on their barcodes. Sequences were binned into operational taxonomic units (OTUs) at a 97% identity threshold with UPARSE method [31]; and representative sequences were mapped to the OTUs. Then taxonomy was assigned to OTUs using RDP classifier against the SILVA taxonomy database [32]. The 16S rRNA gene sequence data were deposited in NCBI Sequence Read Archive under accession number SRP056350.

Rarefaction was carried out at a depth of 10,000 sequences per sample. Species (OTUs) richness, Pielou’s evenness, and Shannon index were calculated for each source soil and each microcosm based on the rarefied OTU tables. Analysis of species persistence in microcosms was performed for species with an initial proportion ≥ 0.0003 (in the source soils). Among the OTUs, those with a final proportion ≥ 0.0001 in a microcosm were assigned as score 1 (persistent) and those < 0.0001, 0 (non-persistent). We excluded OTUs with a proportion < 0.0003 in the source soils from the analysis of species persistence, as the estimation of abundances for the low-abundance OTUs is not very reliable (due to chance effects in the PCR or sequencing processes).

### Bacterial Community Biomass Production

Bacterial community biomass was measured by real-time quantitative PCR (qPCR) of the bacteria 16S rRNA gene V3 region, with the universal primer pair 341-F(5′-CCTACGGGAGGCAGCAG-3′) and 534R (5′-ATTACCGCGGCTGCTGG-3′). Reactions were carried out in a 7500 real-time PCR System (Applied Biosystems by Life Technologies, Singapore) and quantification was based on the increasing fluorescence intensity of the SYBR Green dye during amplification. The PCR reaction volume (20 μL) consisted of 10 μL of *Power* SYBR green PCR Master Mix (Applied Biosystems, USA), 0.5 μL of each 10-μM forward and reverse primer, 7 μL of sterile DNA-free water, and 2 μL of standard and environmental DNA. The program was as the followings: 8-min initial denaturation at 94°C, followed by 45 cycles of 40 s at 94°C, 40 s at 55.7°C, and 40 s at 72°C, and a final 10-min extension at 72°C. Standard curves were obtained using 10-fold serial dilution (10^3^-10^8^copies μL^-1^) of a plasmid containing a full-length copy of soil bacteria 16S rRNA genes. The presence of PCR inhibitors in DNA extracted from soil was estimated by diluting the soil DNA at different levels and mixing a known amount of standard DNA with soil DNA extracted prior to qPCR. Proper dilutions of 100 fold or 150 fold were chosen for qPCR to minimize the qPCR inhibitory. Melting curve and gel electrophoresis analyses were performed to confirm that the amplified products were of the appropriate size. Three no-template blank controls were run for each qPCR assay and no-template controls gave negligible values. Bacterial gene copy numbers were generated using a regression equation for each assay relating the cycle threshold (Ct) value to the known number of copies in the standards. For each DNA extract, qPCR reactions were run in quadruplicate, and the standard deviation of Ct values among four replicates was below 0.3.

### Statistical Analyses

We conducted statistical analyses in the R environment [33]. Differences of the experimental microcosms from the source soils in species diversity indices and biomass production were analyzed using one-sample t test, with the species richness and bacterial abundance data log-transformed (to meet the assumption of homoscedasticity and normal distribution). Relationship between species persistence in each microcosm and their initial proportional abundance was analyzed by the generalized linear model, with a binomial distribution for species persistence data (model as: glm (y ~ x, family = binomial), where y is the response variable and x the explanatory variable); and abundance data were log-transformed. Relationship between species richness and community biomass was analyzed using Pearson’s correlation test.

## Results

### Species Diversity

**Fig 1:**
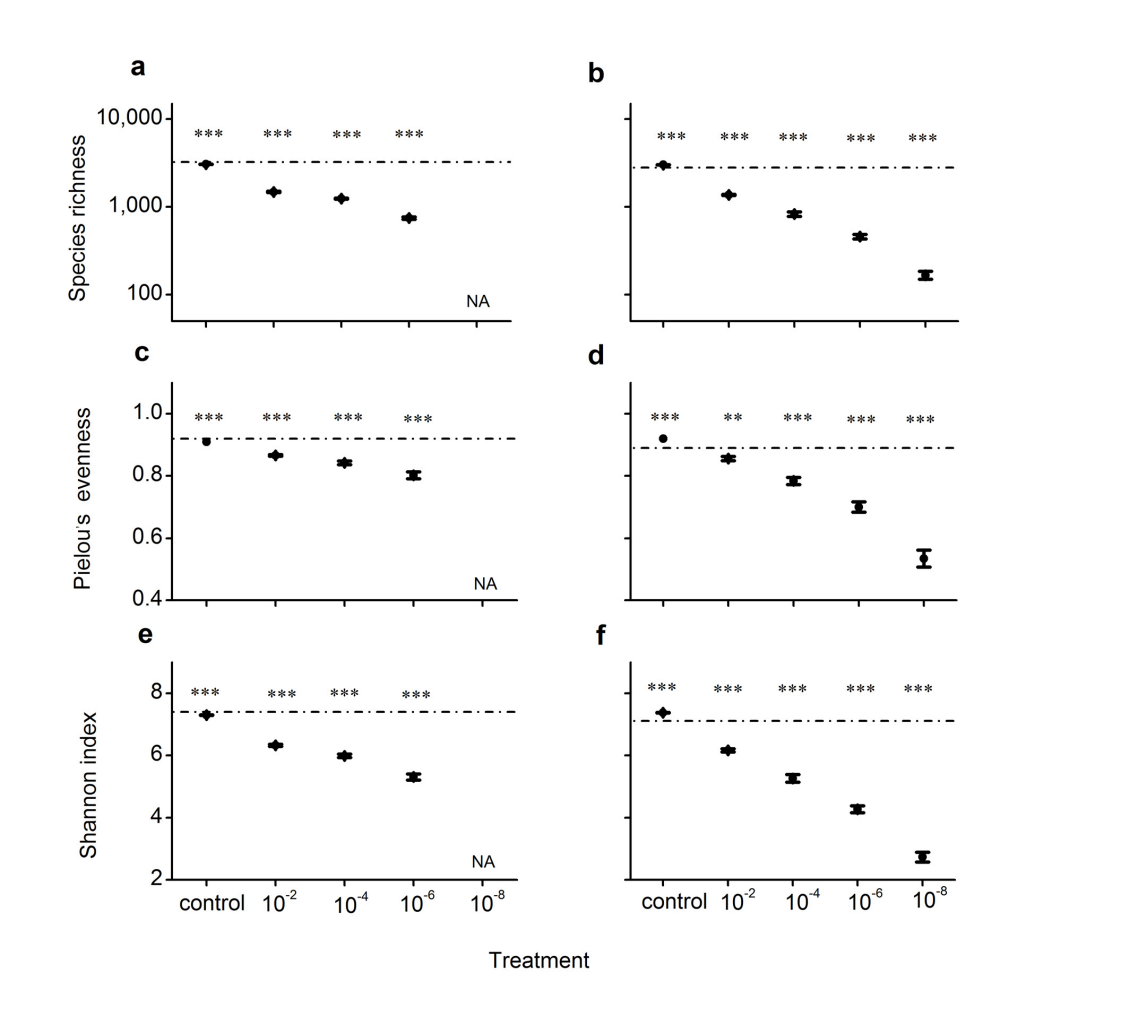
Species (OTUs) richness, Pielou’s eveness and Shannon index of bacterial communities. (a), (c) and (e**),** the sandy soil microcosms. (b), (d) and (f**),** the grassland soil microcosms. The horizontal lines represent the source soils. Asterisks indicate significant departure from the source soils (based on one-sample t test; single asterisk, p < 0.05; double, p < 0.01; triple, p < 0.001). Data show mean ± SE (n = 5).

Three diversity indices, species (OTUs) richness, Pielou’s evenness and Shannon index, were calculated for soil samples based on rarefied OTU tables at a depth of 10,000 sequences per sample. Compared with the source soils, the control microcosms showed slight, although statistically significant, changes in diversity indices. Species richness, Pielou’s evenness, and Shannon index decreased, on average, by 5.7%, 1.1%, and 1.4%, respectively, in the sandy soil microcosms (Fig 1A, 1C and 1E). In the grassland soil microcosms, species richness, Pielou’s evenness, and Shannon index increased by 7.4%, 3.4% and 3.8%, respectively (Fig 1B, 1D and 1F).

The microcosms exposed to, and recovered from, the dilution disturbance showed dramatic decreases in the diversity indices compared with the source soils; and the extent of the decrease became larger with increasing magnitude of dilution disturbance (Fig 1). For instance, in the sandy soil microcosms, species richness declined, on average, by 54%, 62% and 77% in the 10^–2^, 10^–4^ and 10^–6^ dilution treatments, respectively (Fig 1A); in the grassland soil microcosms, species richness declined, on average, by 51%, 71%, 84% and 94% in the 10^–2^, 10^–4^,10^–6^ and 10^–8^ dilution treatments, respectively (Fig 1B).

### Species Persistence

**Fig 2:**
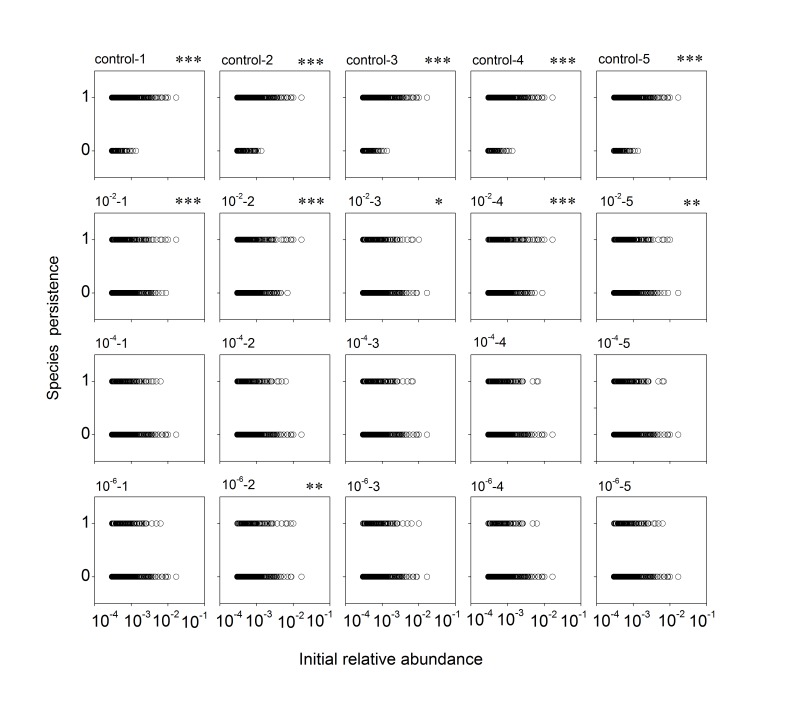
Persistence of species in each sandy soil microcosm as a function of initial abundances. Persistence score of 1 indicates survival, and 0 indicates extinction. Each panel is referred by its microcosm ID; e.g. ‘control-1’ indicate the No. 1 microcosm under the control treatment. Asterisks indicate significantly positive abundance-persistence relationships (single asterisk, p < 0.05; double, p < 0.01; triple, p < 0.001).

**Fig 3:**
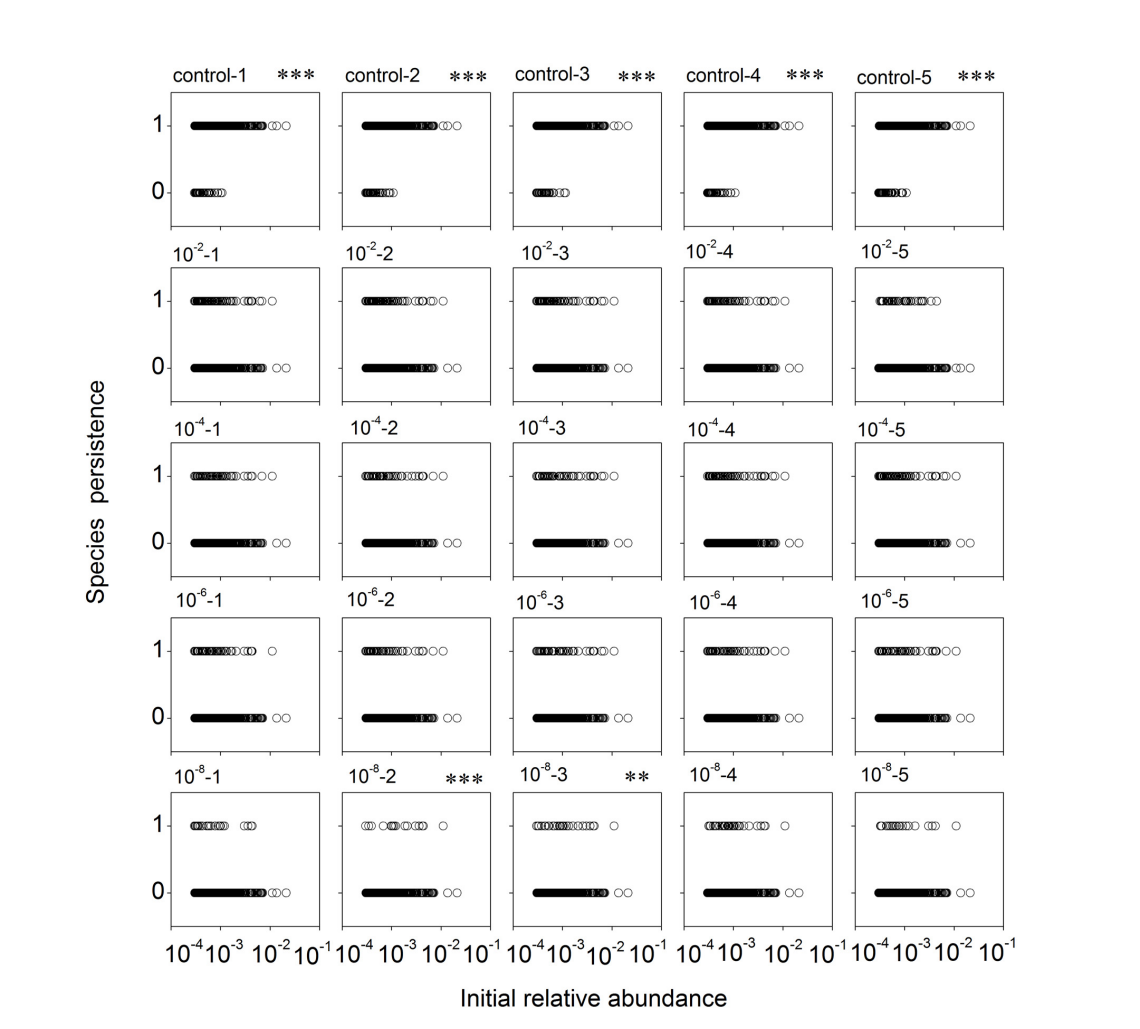
Persistence of species in each grassland soil microcosm as a function of initial abundances. Symbols as in Fig 2.

The persistence of a subset of species, with an initial proportion ≥ 0.0003, was examined. In the control microcosms, species that went extinct (final proportion < 0.0001) were all relatively rare initially; and there was a highly significantly positive relationship between species initial abundance and their chance of persistence (Figs 2 and 3). In the microcosms exposed to the disturbance, more species went extinct. The extinct species included both initially rare and initially abundant species, even the most abundant ones (Figs 2 and 3). The relationship between species initial abundance and chance of survival was positive in a small number of microcosms (six out of 15 microcosms of the sandy soil, and two out of 20 microcosms of the grassland soil) and neutral in the other ones (Figs 2 and 3).

### Community Biomass Production

**Fig 4:**
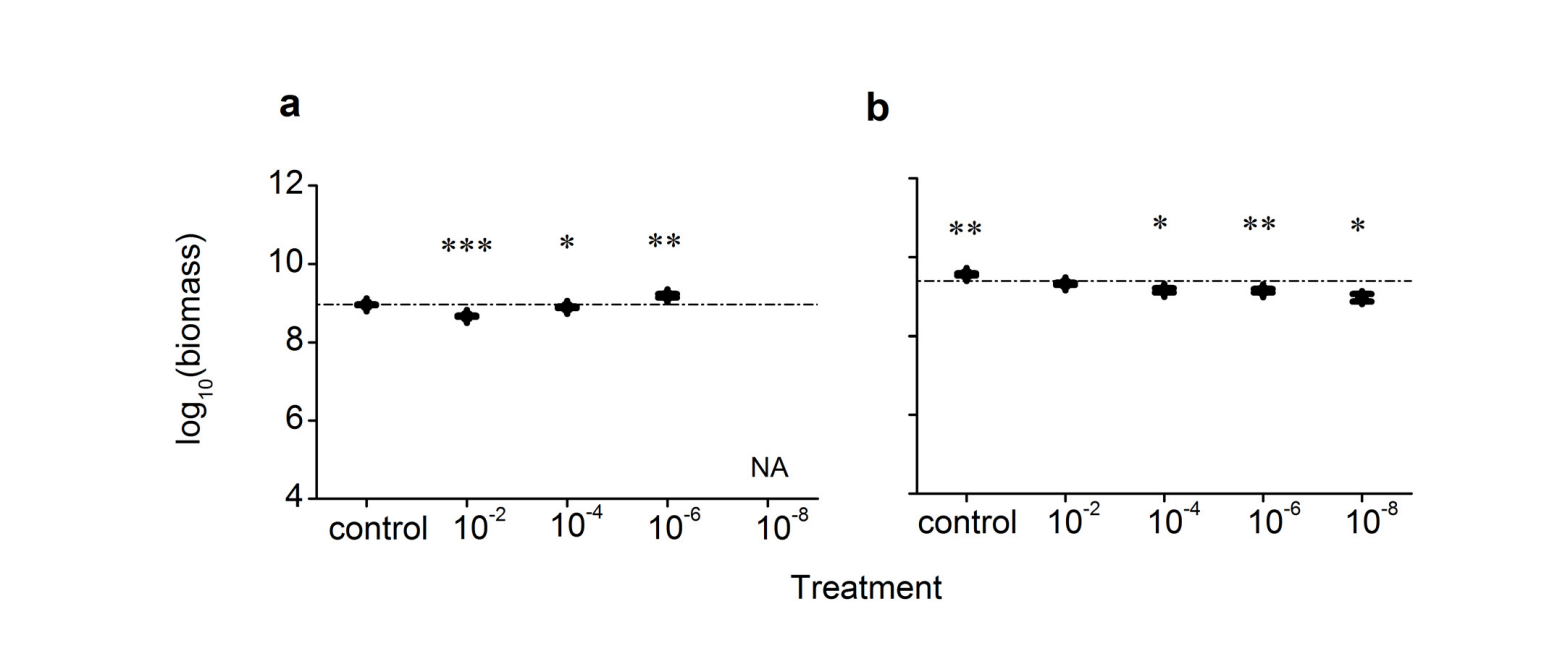
Bacterial community biomass in experimental microcosms. (a), the sandy soil microcosms. (b), the grassland soil microcosms. Asterisks indicate significant departure from the source soils (based on one-sample t test; single asterisk, p < 0.05; double, p < 0.01; triple, p < 0.001). Data show mean ± SE (n = 5).

Bacterial biomass, measured as 16S rRNA gene copy number per gram of dry soil, was 9.2 × 10^8^ in the sandy source soil, and 2.5 × 10^9^ in the grassland source soil. After the recovery incubation, bacterial biomass in microcosms varied among treatment levels, but not in a consistent manner among the two soils. For the sandy soil, bacterial biomass in the control microcosms did not differ significantly from that in the source soil; bacterial biomass in the microcosms under the 10^–2^ and 10^–4^ treatments was, on average, lower than that in the source soil by 50% and 16%, respectively; however, bacterial biomass in the microcosms under the 10^–6^ treatment was, on average, 70% higher than that in the source soil (Fig 4A). There was a not a significant correlation between species richness and biomass production across the microcosms (Pearson’s correlation, r = -0.392, p = 0.087, n = 20). For the grassland soil, the control microcosms had biomass 48% higher than the source soil, and the microcosms under the 10^–2^, 10^–4^, 10^–6^ and 10^–8^ dilution treatments had biomass 14%, 40%, 42% and 59% lower than the source soil (Fig 4B); and there was a significantly positive correlation between species richness and biomass production (r = 0.851, p < 0.001, n = 25).

## Discussion

Neutral processes have been expected to play an important role in structuring microbial communities which are characterized by tremendous diversity [8,10,19,34,35]. Pragmatically, researchers often infer the mechanisms underlying microbial community assembly by examining the biodiversity patterns such as species abundance distribution or distance-decay relationships, as experimental manipulation of microbes is difficult (for instance, it is almost impossible to do *in situ* pair-wise species competition experiments, or to selectively remove specific species from natural environments). Here we address the importance of functional equivalence in soil microbes by studying the changes in both community structure and biomass production in microbes recovering from a disturbance.

Our microcosms were either exposed to a dilution disturbance (treatment microcosms) or not (control microcosms). They may differ little in the physical environment, as sterilization of the treatment microcosms by gamma irradiation may cause minimal changes in soil properties [36]. Rather, they differ primarily in their biotic components. In the treatment microcosms microbes were diluted and thus very rare species were removed and the remaining species had an opportunity for rapid growth during the subsequent incubation. Our microcosm volume was 100 g, and the bacterial abundances in our source soils were near 10^9^ g^-1^. Although our estimation of bacterial abundances by qPCR of 16S rRNA genes may not be very accurate, it would still be safe to suggest that all the species detectable in the source soils by our pyrosequencing (with a frequency ≥ 0.0001) should have survived the low-level dilution procedure (10^–2^ and 10^–4^), and some of them may have been lost in the dilution procedure for the high-dilution microcosms (10^–6^ and 10^–8^). The treatment microcosms suffered a reduction in diversity of species detectable by the pyrosequencing technique (Fig 1). At least for the low-dilution microcosms, the loss of diversity in those initially detectable species should not have been caused by the dilution procedure *per se*; rather, they were likely introduced into the microcosms initially, but then went extinct during the recovery incubation. Previous studies also documented decreases in species diversity in microbial communities that recovered from dilution disturbances, and were aimed at examining the consequences of the diversity loss for ecosystem functions [14,20,21,23]. Intuitively, one may assume that the loss of diversity should reflect the extinction of only low-abundance species, and attribute the changes in ecosystem functions to the loss of the rare species [37]. Our results suggest that this is not the case; not only the very rare species can be removed by the dilution treatment, but also many initially high-abundance species may go extinct during community recovery following the disturbance (Figs 2 and 3).

Our control microcosms showed slight changes in species diversity (Fig 1) and species composition (S2 Fig) during the recovery incubation. The small changes in species composition may reflect both species loss (initially detectable species becoming undetectable) and ‘species gain’ (initially undetectable species becoming detectable). The species lost in the control microcosms were all relatively rare initially (Figs 2 and 3). This result does not falsify either neutral or adaptation mechanisms. The stability of the abundant species may be explained by better adaptation to the environment. Alternatively, neutral processes may have been important. They could have led to the loss of some rare species and the initially abundant species persisted simply because they were less vulnerable to demographic stochasticity. Some of them may have gone extinct if our experiment had been run for a longer period.

For the patterns observed in the treatment microcosms (that recovered from the dilution disturbance), the adaptation hypothesis may provide much better explanations. First, increasing dilution treatment led to progressive decreases in species diversity, and progressive dissimilarity in species composition, compared with the source soils (Fig 1; S2 Fig), consistent with earlier studies [14,20]. As mentioned above, species detectable in our source soils (with a frequency ≥ 0.0001) should all have been introduced into the microcosms, at least for the low-dilution microcosms (10^–2^ and 10^–4^), and the loss of diversity in the detectable species in those microcosms is likely to occur during the recovery incubation. Drift processes might be responsible for some extinction events: when the community sizes were reduced by the dilution treatment, more species may go extinct by chance. However, the diversity loss was of very large magnitude (> 50%). Random extinction due to drift processes is unlikely to occur so rapidly [2,38]. Second, in the treatment microcosms both initially rare and initially abundant species (even the most abundant ones) may go extinct; in a majority of the treatment microcosms there was not a positive relationship between species initial abundance and their chance of persistence (Figs 2 and 3). This can only be explained by adaptation mechanisms determining the community structure, as a positive abundance-persistence relationship should be an inevitable prediction for neutral processes driving species extinction. It is noteworthy that some species detectable in our source soils might have been lost during the experimental dilution procedure for the high-dilution (10^–6^ and 10^–8^) microcosms. Such species ‘extinction’ events should be biased to initially low-abundance species, but not initially abundant species. Therefore, the absence of a species abundance-persistence relationship in most of the microcosms recovering from the disturbance cannot be a result of the dilution procedure *per se*. Besides, the large differences between the control and the treatment microcosms in species richness and composition also suggests that contamination by environmental microbes during the recovery incubation should have been negligible (as contamination can lead to higher similarity in species composition among microcosms)

We may make some inference about the specific adaptation mechanisms involved in organizing our bacterial communities. There were two major changes imposed on the bacterial communities by the dilution treatment: reduced community sizes (and thus an opportunity for rapid recovery growth), and loss of very rare species (and thus loss of some interspecific interactions). The replacement of some initially abundant species by certain initially low-abundance species during the recovery growth might reflect the operation of *r*/*K* selection in soil bacteria [25,26,27,28]. In our source soils the abundant species might be those with high carrying capacity but low intrinsic growth rate, and during the recovery growth following the dilution treatment certain species that were previously rare, but capable of rapid growth, could become dominant. Alternatively, the initially abundant species may not be those perfectly adapted to the physical environment; instead, their success depended on adaptation to the biotic environment. For instance, they may be those benefiting from mutualistic or commensal relationships with other species [39,40], or better defended against predators than their competitors [41]. The dilution treatment could reduce the efficacy of some relationships by decreasing the absolute abundances of species, or even completely remove the species interactions that involved very rare species. Both the r/K selection and the species interactions loss hypotheses can explain the reduction of some initially abundant species in the disturbed microcosms. However, the dramatic decrease (> 50%) in species diversity may be better explained by the species interactions loss hypothesis. The loss of positive species interactions or predatory interactions can lead to further decreases in species diversity [42,43], but there is no good reason to expect that replacement of *K*-species by *r*-species be accompanied by a very large decrease in overall species diversity.

The different specific mechanisms also differ in their predictions for the responses of community biomass production to changes in species composition, which depends on the compensatory dynamics following species loss [14,20,21,44]. In a scenario of replacement of *K*-species by *r*-species, the community biomass should decline as the *r*-species have lower carrying capacities. In cases where species are lost due to a reduction in positive species interactions, community biomass may generally decrease. When species composition changes as a result of loss of prey-predator interactions, however, community biomass may increase, not only because the consumption of biomass on the community level might decrease, but also as a result of more defended species being replaced by more competitive species (if there was a competition-defence trade-off as generally assumed). A progressive decrease in community biomass with increasing dilution disturbance was observed for our grassland soil microcosms (Fig 4B), consistent with the expectation of *r*/*K* selection or loss of positive interactions altering community structure. However, this is not the case for the sandy soil. The sandy soil had lower bacterial abundance compared with the grassland soil. The 10^-8^ dilution treatment might have been a very strong disturbance to the sandy soil, suggested by the fact that microcosms recovering from this treatment had very low density of culturable bacteria (S1 Fig) and PCR was unsuccessful for DNA extracted from those microcosms. Sandy soil microcosms recovering from 10^–2^ and 10^–4^ treatments had lower biomass compared with the source soil. However, sandy soil microcosms recovering from 10^–6^ treatment had higher biomass (Fig 4A). One possibility is that the 10^–6^ treatment significantly reduced the impact of predators on bacteria, relative to the 10^–2^ and 10^–4^ treatments.

Our two soils differed in physical properties (texture, moisture content, and probably resource availability) and bacterial diversity. Patterns observed in microcosms of the two soils are in agreement for a rejection of the neutral hypothesis, although subtle differences may exist in the importance of specific species interactions for community assembly. In summary, adaptation mechanisms, but not neutral processes, appear to play a predominant role in organizing our soil bacterial communities. The abundant species in our source soils are likely to be those benefiting from species interactions such as facilitation or predation.

## Supporting Information

S1 Fig Abundances of culturable bacteria in a subset of microcosms during the recovery incubation. (a), the sandy soil microcosms. (b), the grassland soil microcosms. Bacterial biomass should have reached a plateau before week 5, except for the sandy soil microcosms under the highest dilution disturbance (in which culturable bacteria were not detectable, < 10^2^ g^-1^, before week 7, but reached to stable levels after week 10)
(TIF)

S2 Fig Nonmetric multidimensional scaling (NMDS) plots based on the between-sample Bray-Curtis dissimilarities. (a), the sandy soil microcosms. (b), the grassland soil microcosms. The analyses were done based on rarefied OTU tables at a depth of 10,000 sequences per sample. The differences in overall community composition between each pair of microcosms were measured by the Bray-Curtis dissimilarity index, based on which nonmetric multidimensional scaling (NMDS) plots were derived. These analyses were carried out using the vegan package in the R environment.
(TIF)
