# Supplementary figures and images for: Patterns in Species Persistence and Biomass Production in Soil Microcosms Recovering from a Disturbance Reject a Neutral Hypothesis for Bacterial Community Assembly
Source: PLoS One. 2015 May 11;10(5):e0126962. doi: 10.1371/journal.pone.0126962 (PMC4427283; doi:10.1371/journal.pone.0126962)

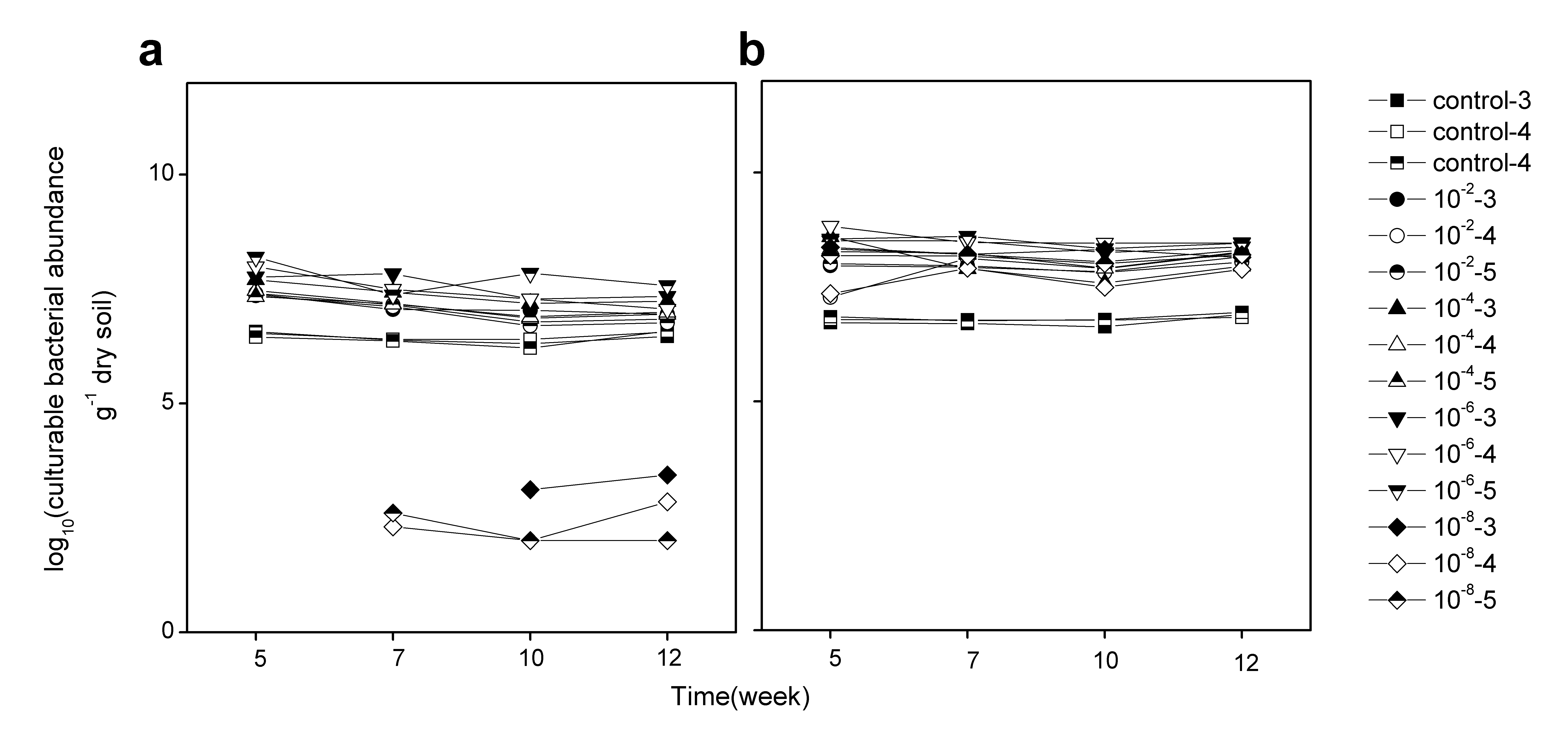

Supplement: S1 Fig — (a), the sandy soil microcosms. (b), the grassland soil microcosms. Bacterial biomass should have reached a plateau before week 5, except for the sandy soil microcosms under the highest dilution disturbance (in which culturable bacteria were not detectable, < 102 g-1, before week 7, but reached to stable levels after week 10) (TIF) [file pone.0126962.s001.tif]

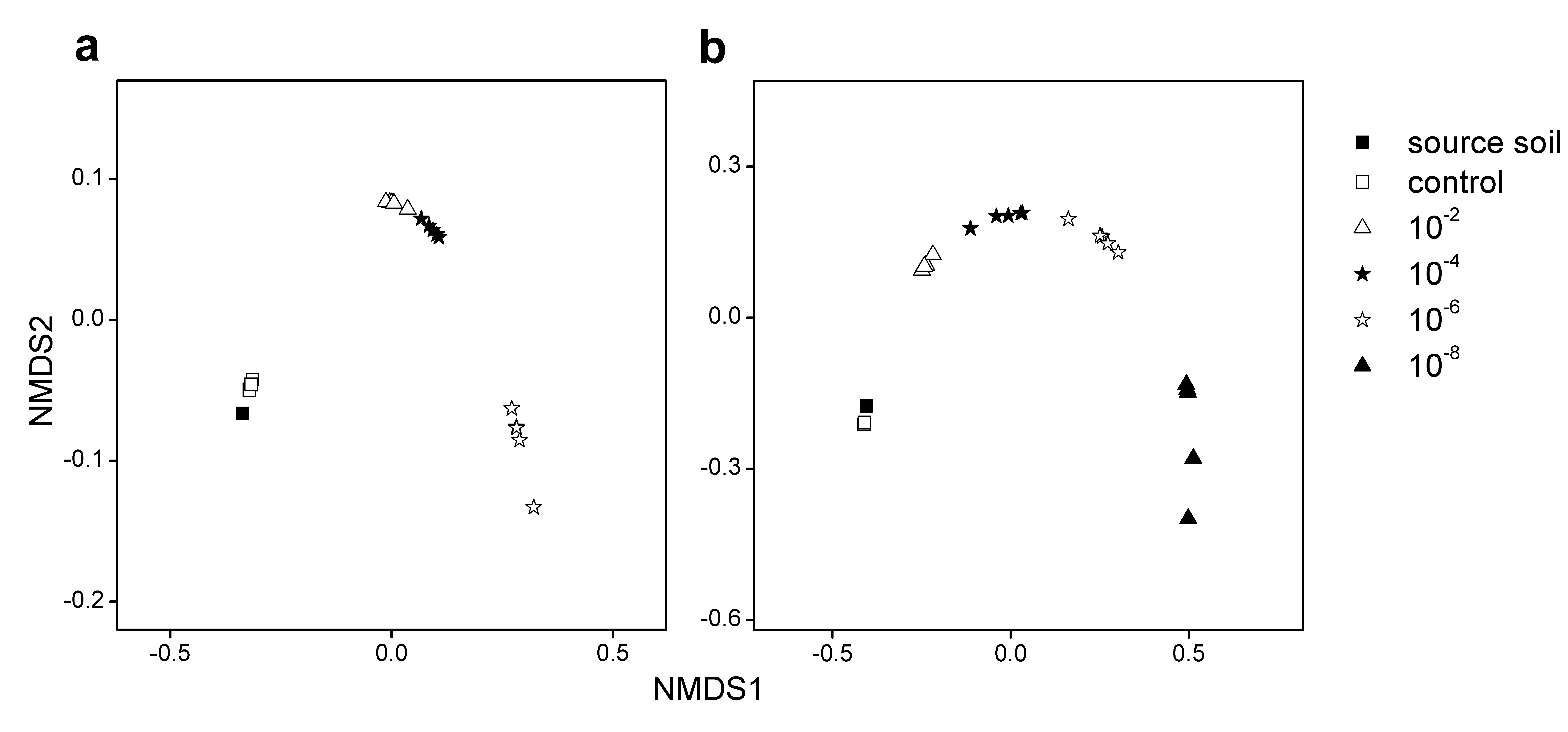

Supplement: S2 Fig — (a), the sandy soil microcosms. (b), the grassland soil microcosms. The analyses were done based on rarefied OTU tables at a depth of 10,000 sequences per sample. The differences in overall community composition between each pair of microcosms were measured by the Bray-Curtis dissimilarity index, based on which nonmetric multidimensional scaling (NMDS) plots were derived. These analyses were carried out using the vegan package in the R environment. (TIF) [file pone.0126962.s002.tif]
